# Vat photopolymerization of multifunctional fresnel lenses for ocular management

**DOI:** 10.3389/fbioe.2024.1464129

**Published:** 2024-10-08

**Authors:** Murad Ali, Muhammed Hisham, Rashid K. Abu Al-Rub, Haider Butt

**Affiliations:** ^1^ Department of Mechanical and Nuclear Engineering, Khalifa University of Science and Technology, Abu Dhabi, United Arab Emirates; ^2^ Advanced Digital & Additive Manufacturing (ADAM) Center, Khalifa University of Science and Technology, Abu Dhabi, United Arab Emirates

**Keywords:** fresnel lens, 3D printing, vat photopolymerization, color vision deficiency, myopia, conventional lenses

## Abstract

In this study, multifunctional Fresnel lenses were explored as a potential solution for correcting vision in patients with color vision deficiency (CVD) and high myopia. Current studies have primarily focused on color vision correction through the 3D printing of glasses and contact lenses. However, the potential of 3D-printed multifunctional devices, such as Fresnel lenses, goes beyond addressing a single vision correction issue. For this study, computer-aided design (CAD) model of Fresnel lens with high diopter based on constant height configuration was developed. The CAD model was successfully fabricated using vat photopolymerization 3D printer, employing laboratory-prepared transparent HEMA resin. The resin was modified with two Atto dyes (565 nm and 488 nm), known for their ability to filter out problematic wavelengths (400–500 nm and 540–580 nm) to address color vision deficiency. The printed lenses were characterized by their chemical, physical, and optical properties using various characterization techniques. The focusing performance was evaluated using focal length measurements, and the results obtained were less than 2 mm deviation from the design value, having the potential to assist in higher myopic vision correction. The resulting optical spectra were compared with commercial glasses, revealing close agreement for CVD correction. These results expand the potential applications of multifunctional Fresnel lenses in ophthalmology, demonstrating their effectiveness as vision-correcting lenses and imaging systems.

## 1 Introduction

**FIGURE 1:**
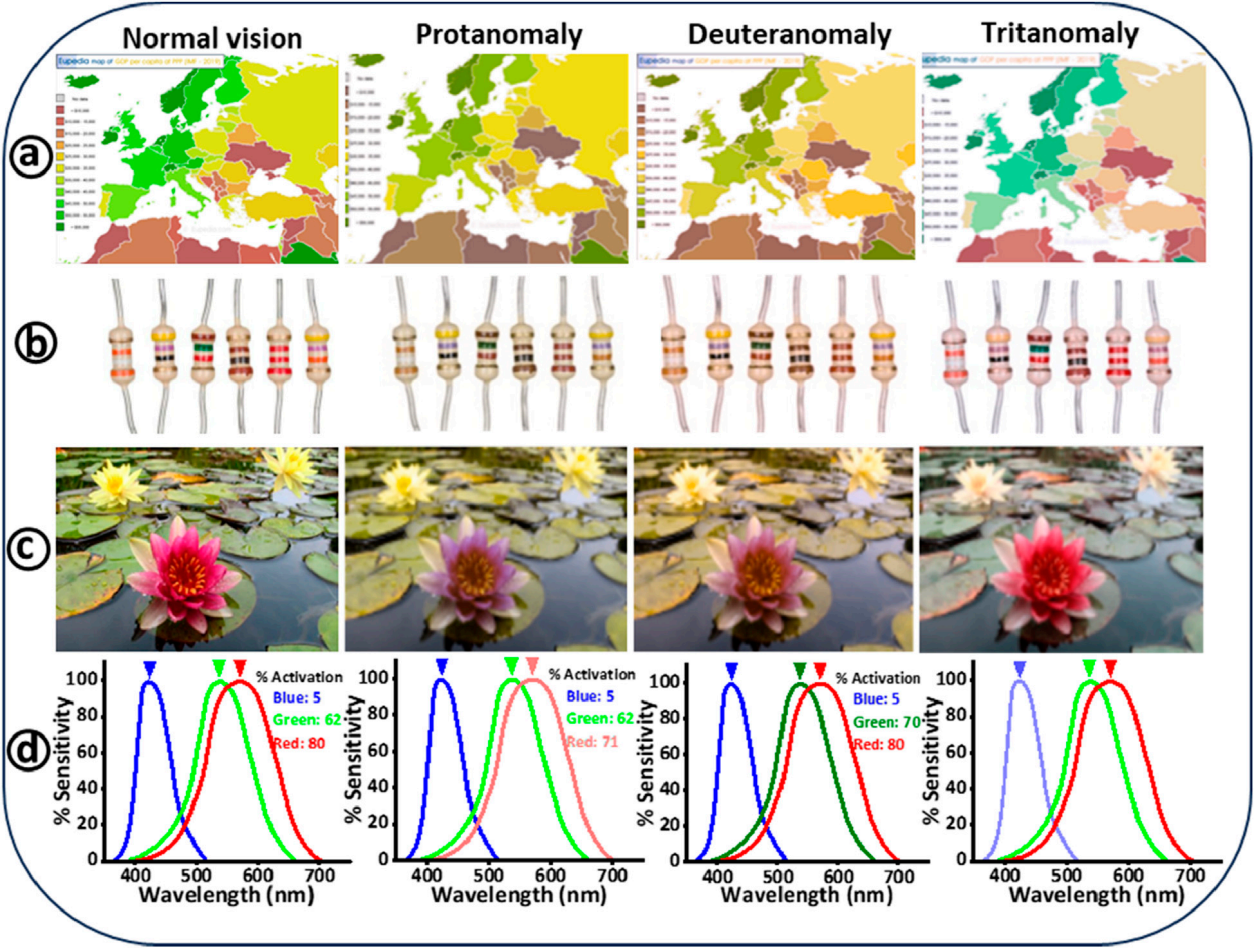
Illustrates how color images appear to individuals with normal vision compared to those with color blindness. The images on the left show the original colors, while on the right, simulations from the colorblindness simulator’s website depict how these images appear to individuals with different types of color vision deficiencies: protanomaly, deuteranomaly, and tritanomaly. **(A)** Displays a map depicting Europe’s gross domestic product (GDP) *per capita* in 2019 (Maciamo). **(B)** Digital images showing various electric resistors, with each device’s resistance indicated by color-coded strips (Bracho Garcia, 2023). **(C)** Depicts an image of springtime water lily flowers (Lin et al., 2019). **(D)** Demonstrates the activation of photoreceptor cells at 520 nm, presenting the differences in perception between normal vision, protan, deutran, and tritan color vision deficiencies (Alam et al., 2022b).

Color distinction is fundamental to how we perceive and interact with the world, influencing everything from daily tasks to professional activities. Color vision deficiency (CVD), commonly known as color blindness, disrupts this process, affecting approximately one in 12 men and one in 200 women globally (Alamoudi et al., 2021). This condition arises from genetic or acquired deficiencies in the photopigments of the eye’s cone cells, which are crucial for color perception colors (Alam et al., 2022c). Individuals with CVD face numerous challenges, including difficulties in interpreting maps (Maciamo), and technical color-coded information resisters (Bracho Garcia, 2023), as shown in Figure 1. Moreover, recognizing color differences in various environments, such as greenery, which can evoke emotional responses (Lin et al., 2019) (Figures 1A–C). These challenges extend to practical issues like identifying traffic lights, LED indicators, ripe fruits, and sports jerseys (Alam et al., 2022b), potentially leading to feelings of depression due to a diminished ability to appreciate the full spectrum of colors.

The mechanism of color detection in the human eye relies on cone cells, which are concentrated in the fovea centralis of the retina (Welby et al., 2017). These cone cells are situated in an area of the retina near the back of the eye called the fovea centralis, covering an area of 0.3 mm^2^ (Dua et al., 2011). These cells are categorized as short (S), medium (M), and long (L) cones, corresponding to blue, green, and red light, respectively (Deeb, 2006). Normal color vision requires the brain to process signals from all three cone types (Figure 1D), but individuals with CVD typically lack or have dysfunctional cone cells, affecting their color perception. Studies show that approximately 7%–8% of males and 0.4%–0.5% of females are affected by congenital CVD (Salih et al., 2020), limiting their participation in professions like aviation and transportation, where accurate color differentiation is critical for safety (Bracho Garcia, 2023). CVD is classified into three levels: anomalous trichromacy, dichromacy, and monochromacy (Simunovic, 2009). Anomalous trichromacy includes protanomaly (faulty L-cones), deuteranomaly (faulty M-cones), and tritanomaly (faulty S-cones). Dichromacy involves the absence of one type of cone, leading to conditions such as protanopia, deuteranopia, and tritanopia. Monochromacy, the rarest form, results in either complete color blindness (achromatopsia) or limited blue cone vision. The most common forms of CVD are the red-green deficiencies (protans and deutans), with blue-yellow deficiencies (tritans) being less common. High myopia, another significant ocular condition, is defined by severe nearsightedness requiring correction of −5 diopters or more. High myopia can be corrected optically using glasses, contact lenses, or refractive surgery. However, it's important to note that while these methods improve vision, they do not address the underlying structural changes in the eye associated with high myopia (Haarman et al., 2020; Williams and Hammond, 2019). This condition is increasingly recognized as a global health concern due to its association with complications like glaucoma, macular degeneration, and retinal detachment (Haarman et al., 2020; Kong et al., 2021). The prevalence of high myopia is rising rapidly, with projections suggesting it could affect nearly 10% of the global population by 2050 (Fricke et al., 2018; Zhang et al., 2024). This trend underscores the urgent need for effective management strategies to mitigate the impact of high myopia.

Despite the lack of a definitive cure for CVD, recent advancements in technology have provided new management options. Gene therapy remains in experimental stages primates (Simunovic, 2009; Zhao et al., 2019), while specialized glasses and contact lenses with color filters offer some relief for CVD patients (Simunovic, 2009; Zhao et al., 2019). Emerging technologies like 3D-printed medication labels are also being explored to assist with color recognition (Wong et al., 2020). The concept of selective color filters based on visual aids was initially introduced by Schornack, who asserted that CVD patients could distinguish colors through their usage (Schornack et al., 2007). Companies like EnChroma and Chromagen lead the industry in developing wearables for CVD, although customizing these products to individual needs remains challenging. Additionally, advances in additive manufacturing, particularly 3D printing, have opened new possibilities for creating personalized optical devices, including tinted glasses, contact lenses, and Fresnel lenses (Alam et al., 2022b; Alam et al., 2022a; Alam et al., 2022c; Ali et al., 2022; Lee et al., 2020).

Historically, Fresnel lenses were primarily used in solar collector applications (Watson and Taminger, 2018), but their lightweight design, lower production costs, and reduced transmission loss compared to conventional lenses have expanded their applications (Ali et al., 2021; Awasthi et al., 2020; Pham et al., 2018). Today, they are used in various fields, including 3D viewing systems (Hsu et al., 2021), magnification (Wang et al., 2021), and infrared imaging systems (Claytor and Claytor, 2003; Sugiyama et al., 2017). Interest in their ophthalmological applications has grown (Elsherif et al., 2021), leading to the development of liquid crystal-based Fresnel lenses for near-eye treatments (Zhang, 2020). Research is ongoing to assess their effectiveness in treating conditions like hemianopia (Perez and Jose, 2003), presbyopia (Bryant et al., 2018; Fan et al., 2003), and strabismus (Kaur and Gurnani, 2023). However, the fabrication of micro-Fresnel lenses poses challenges due to their small size and the need for precise optical surfaces (Nachmias et al., 2009; Senzaki et al., 2022). Conventional methods like casting and injection molding offer some solutions, but they limit design flexibility (Ali et al., 2022).

In this study, we used vat photopolymerization 3D printing to fabricate functional Fresnel lenses for managing CVD and correcting myopic disorders. We prepared a transparent photocurable resin mixed with two specific wavelength-filtering dyes (Atto 565 and Atto 488) to create the desired printing resin. This resin was used to print lenses at three different dye concentrations (1.5%, 2.5%, and 5%), which were then characterized for their morphological, structural, and optical properties. We compared their optical characteristics with those of commercially available glasses designed for CVD management.

## 2 Materials and methods

### 2.1 Materials

The components of the photocurable resin, such as 2-hydroxyethyl methacrylate (HEMA, 97%), polyethylene glycol diacrylate (PEGDA, 98%) having a molecular weight of 2000 Da, and diphenyl phosphine oxide (TPO, 99%) were procured from Allplace, co., Ltd. (Shandong, China) and used as is without further purification. Additionally, Atto dyes, specifically Atto 565 and Atto 488, were obtained from Sigma Aldrich. Isopropyl alcohol (IPA) and dimethyl sulfoxide (DMSO) were supplied by Merck KGaA (Darmstadt, Germany). The transparent resin based on HEMA was subsequently modified using Atto 565 and Atto 488 dyes to achieve specific wavelength filtering capabilities. The experiments also utilized a commercially available smooth, transparent plastic film composed of polyvinyl chloride (PVC) and deionized (DI) water.

### 2.2 Preparation of 3D printing resin

**FIGURE 2:**
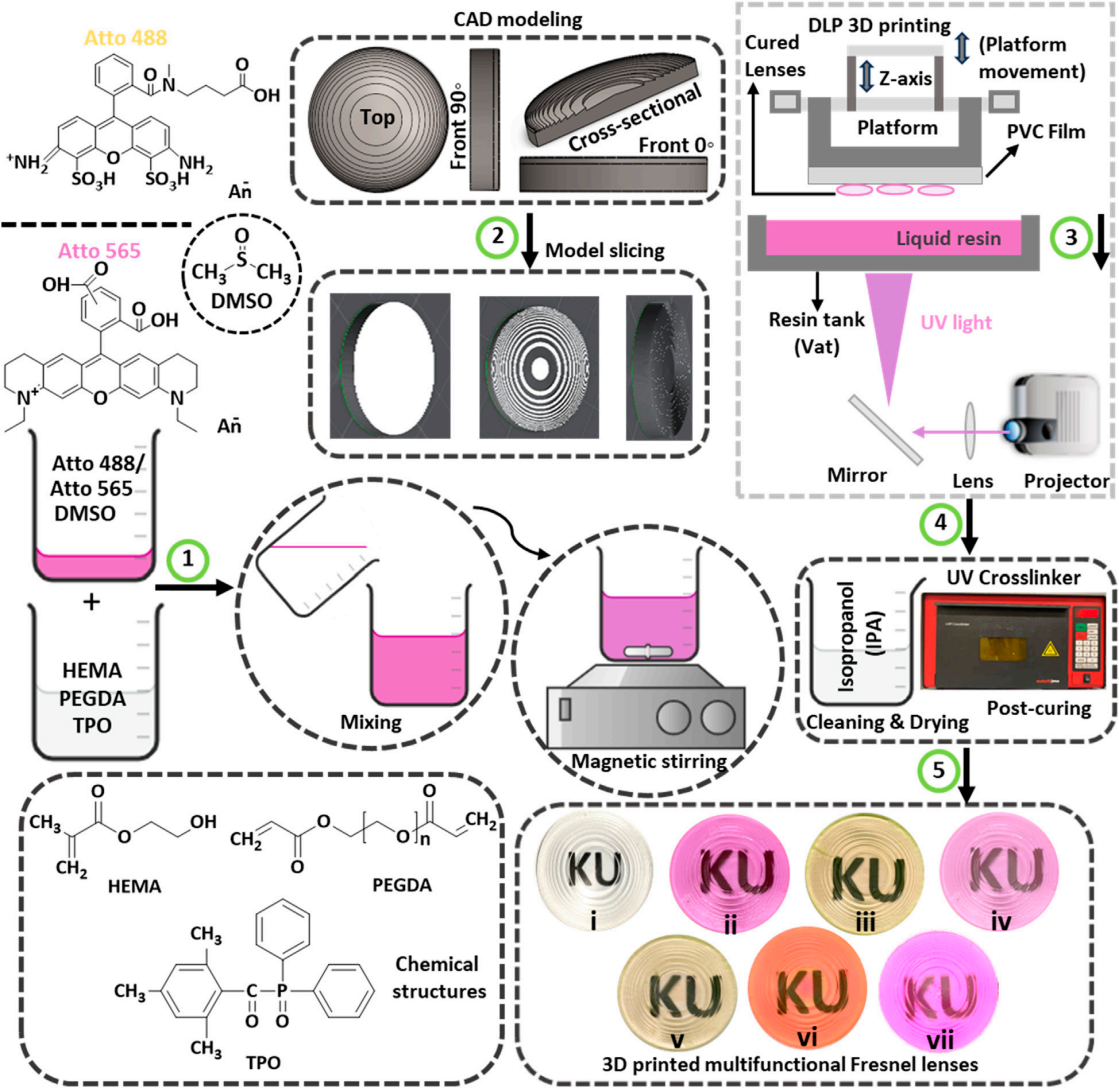
Presents a schematic illustration detailing the 3D printing process for transparent and multifunctional Fresnel lenses, which is outlined in five different steps: (1) The first step involves the representation of chemical structures and the mixing of resin components. This encompasses the representation of chemical structures, the exhibition of their mixing process, and the utilization of magnetic stirring to attain homogenized 3D printing resins. (2) The second step encompasses CAD modeling and slicing of the designed Fresnel lens. (3) The third step provides a schematic illustration of the UV light-based 3D printing process. (4) In the fourth step, cleaning of 3D printed lenses using IPA is carried out, followed by post-curing in a UV chamber. (5) The final step represents digital images of 3D printed Fresnel lenses, including i) transparent, ii) fully pink, iii) fully yellow, iv) transparent base with pink features, v) transparent base with yellow features, vi) pink base with yellow features, and vii) yellow base with pink features.

To begin, the photocurable resin was formulated by combining HEMA (monomer) and PEGDA (crosslinker) in a 1:1 volume ratio, followed by the addition of 2.5% by weight of TPO (photoinitiator). HEMA-based polymer is flexible and known for its reported biocompatibility in previous studies (Zhang et al.). On the other hand, PEGDA is a long-chain, flexible polymer that aids in crosslinking HEMA and is well-suited for the formulation of photopolymerizable polymers (Alam et al., 2022a). TPO was chosen as the photoinitiator due to its broad sensitivity to UV light (385–420 nm), and this selection aligns with the requirements of the 3D printer, which necessitates a photoinitiator with a wide range of curing wavelengths. The chemical structures of HEMA, PEGDA, Atto 565, Atto 488, and DMSO are provided in Figure 2 (Step 1). This process involved mixing 50 mL each of HEMA and PEGDA to prepare a 100 mL solution, followed by the addition of 2.5 g of TPO. The resulting mixture was covered with aluminum foil to prevent exposure to ambient light and stirred on a magnetic plate for 20 min to ensure thorough dissolution. Next, Atto dyes (565 and 488), each in powdered form (1 mg), were mixed with 1 mL of DMSO to produce homogeneous liquid dye solutions suitable for mixing with HEMA resin. Subsequently, the previously prepared HEMA resin was tailored using three distinct concentrations—1.5%, 2.5%, and 5% by volume of each dye (Figure 2, Step 1). These varied concentrations of dyes were categorized based on the dye type and the volume percentage employed. For instance, “Atto 565%–1.5%” indicates a transparent liquid resin modified with 1.5% volume of Atto 565 dye, and so forth. The same nomenclature convention applies to “Atto 488%–1.5%”, denoting a transparent liquid resin modified with 1.5% volume of Atto 488 dye, and so on.

### 2.3 Preparation of fresnel lens CAD model

A Fresnel lens was designed using the computer-aided design (CAD) software SolidWorks, adopting a constant height configuration with concentric features. Careful consideration was given to the dimensions of the lens, accounting for the resolution of the 3D printer (including layer thickness) and the constraints of the custom optical experimental setups. Consequently, a lens with a 30 mm diameter was chosen, comprising 15 annular rings. The height of the lens features or annular rings acting as individual prisms was chosen as 0.5 mm. To facilitate the fabrication of a flat Fresnel lens, a lens base thickness of 1.5 mm was incorporated to support the lens features. The resulting CAD model depicted a lens standing at approximately 2 mm in height with a focal length of 60 mm (∼17 diopter). Various views of the prepared CAD model including top, front at 0°, front at 90°, and cross-sectional views are shown in Figure 2 **(Step 2)**. Subsequently, the CAD model of the Fresnel lens was saved in stereolithography (.stl) format and transformed into geometric code (g-code) using the 3D printer’s slicing software (Lychee slicer 5.3.4). Lychee Slicer^®^ is a robust software solution specifically tailored for preparing 3D models for resin-based printers based on stereolithography (SLA), digital light processing (DLP), and liquid crystal display (LCD) 3D printing techniques. For reference, the schematic illustration of the designed Fresnel lens can be found in Supplementary Figure 1 (supporting information, SI), showcasing the half-lens and the *i*th zone in Supplementary Figure 1A, B, respectively.

### 2.4 3D printing of tinted fresnel lenses

The additive manufacturing of transparent and tinted Fresnel lenses was conducted using an open-source LCD-based 3D printer; namely, the Wanhao Duplicator D8. The 3D printer utilizes a UV LED array as its light source, emitting light at 405 nm with a power density of 3.15 mW/cm^2^ to illuminate the LCD screen. The LCD printer employs a built-in light projector to cure the respective resin material, illuminating the transparent Teflon vat (resin tank). It is worth noting that the Teflon vat has a lifespan 10 times longer than traditional PDMS vats. During the printing process, when light reaches the resin tank, an entire layer corresponding to the specified layer thickness is exposed and cured. The cured resin layer adheres to the printing head or platform and ascends to create space for the subsequent layer. As each layer in the model is sequentially cured, a 3D-printed part is produced at the end. Due to the sensitivity of this 3D printing process to external light, a UV-protective glass hood covers the resin tank to prevent interference from ambient light. The schematic illustration of the 3D printing process is shown in Figure 2 (Step 3). The layer thickness can be adjusted within the range of 35–100 µm based on the specific model requirements. The printer offers a maximum printable area of 192*120*180 mm, with x, y, and z accuracy of 0.075, 0.075, and 0.010 mm, respectively. Additionally, the 3D printing speeds for this printer can vary between 20–30 mm/h to produce the desired parts. Given the significant impact of 3D printing parameters on the properties of the manufactured parts, optimization efforts focused on curing time and layer thickness were undertaken to achieve the desired optical properties. The specific printing parameters used are outlined in Table 1.

**TABLE 1:** Optimized 3D printer parameters utilized for printing Fresnel lenses.

Printing parameters	Specifications
Layer thickness	35 µm
Curing time	Burn layer 75 s, norma layer 30 s
Number of burn layer	5
Lifting speed	45 mm/min
Lifting distance	7 mm
Retracting speed	45 mm/min
3D printing support structure	No
Printed surface	Polyvinyl chloride film, 1 mm thick

The selection of layer thickness and curing time was carefully guided by the 3D printer’s capabilities, the optical performance requirements of the printed lens features, and empirical testing. For the Fresnel lens, which has a feature height of 0.5 mm and a base height of 1.5 mm, a thinner layer was preferred to accurately print these features and minimize the stair-step effect. After testing, a final layer thickness of 35 µm was chosen as the optimal compromise between manufacturing feasibility and lens efficiency, balancing optical performance and structural integrity. The curing time, determined by the custom resin used and further testing, was optimized at 30 s per normal layer to ensure complete polymerization without causing thermal deformation. The curing time for the burn layer was set slightly more than twice that of the normal layer (75 s) to ensure proper adhesion of the printed lens to the print bed during the 3D printing process.

The printing process was interrupted at designated time intervals to produce multimaterial Fresnel lenses. This interruption halted the printing process, allowing the build plate to rise for manual access to the partially printed sample. Manual removal of liquid resin from the vat was carried out, and a different resin, distinguished by a distinct dye, was introduced. The printed sample adhering to the build plate underwent thorough cleaning to eliminate any residue of the previous resin. This cleaning step was crucial to prevent the blending of previously used material with the new resin added to the vat. Careful cleaning was imperative to avoid detachment of the sample from the build plate, and the process involved gently running isopropyl alcohol (IPA) over the sample followed by careful drying. Subsequently, the printing process was resumed, resulting in the fabrication of the sample using two distinct dyed resins. At the end of each printing, the respective samples were cleaned with IPA to remove any residual uncured resin, followed by a drying process for 30 min at room temperature. The dried samples were then kept in UV crosslinker for an additional 2 min to ensure complete curing of the samples as shown in Figure 2 (Step 4). Finally, the digital images of the transparent, tinted, and multimaterial tinted Fresnel lenses are shown in Figure 2 (Step 5). These printed lenses can be recognized as: (i) transparent, (ii) fully pink, (iii) fully yellow, (iv) transparent base with pink features, (v) transparent base with yellow features, (vi) pink-based with yellow features, and (vii) yellow-based with pink features.

### 2.5 Materials characterization

The chemical structures and state of photopolymerization of UV resin components before and after printing was carried out using Fourier transform spectroscopy (FTIR) (Kumar et al., 2017). For this purpose, the spotlight 200 FTIR microscopy system from Perkin Elmer was employed to capture transmission spectra of resin components within 500–4,000 cm^-1^ range. The instrumental spectral range was set at 4 cm^-1^. Additionally, the transmission mode was chosen, and 32 scans were employed to acquire FTIR spectra. An optical microscope (Axio Scope 1) manufactured by ZEISS was employed to examine the morphological and optical transmission properties. The transmission properties of the 3D-printed Fresnel lenses were assessed by using a UV-vis Spectrometer (USB 4000+, Ocean Optics) with a detection range of 400–1,100 nm in combination with an optical microscope. The transmission spectra of resin components before and after printing were recorded in the form of transmitted intensity (%) with respect to the wavelength (nm). The printing process quality and integrity were evaluated by scanning electron microscope (SEM). For this purpose, the JEOL JSM-7610F model was used for analyzing surface morphology. The SEM operated with an accelerating voltage of 5 kV and a working distance of 8 mm. To facilitate SEM analysis, a thin layer of gold (approximately 10 nm) was coated onto the sample using a sputtering machine (JEOL JEC-3000FC). For observing the internal surface of the 3D printed lens, a cryogenic fracturing method was employed. This involved subjecting the sample to liquid nitrogen, causing it to fracture in a brittle manner followed by a cross-sectional examination. The surface roughness of the printed sample was investigated using atomic force microscopy (AFM). AFM from Asylum Research was utilized in tapping mode (ac-mode) with drive frequency of 298 kHz. A 5 by 5-micron area was scanned at 0.80 Hz. Furthermore, other parameters in tapping mode used were drive amplitude of 125.7 mV, setpoint of 805.3 mV, and integral gain of 14. The focusing performance was evaluated using a customized optical setup with three different lasers: blue (450 nm), green (532 nm), and red (650 nm).

## 3 Results and discussion

Hydrogels consist of hydrophilic polymer networks created through either physical or chemical interactions (Chung and Park, 2009). In physical hydrogels, the networks are interconnected through molecular entanglements strengthened by secondary forces, whereas chemical hydrogels typically feature networks crosslinked through covalent bonds (Pharmacodyn. and 1963). Furthermore, the literature indicates that the mechanical properties of HEMA hydrogels are contingent upon their structure, particularly porosity, and the extent of hydrophilicity. The synthesis of chemical hydrogels through photo-crosslinking involves a three-step process encompassing initiation, propagation, and termination of free radical reactions induced by UV light. In this work, TPO as a photoinitiator was excited by 405 nm UV illumination from the 3D printer source, the excited TPO attacks the C=C bonds of PEGDA acting as a crosslinker and leading to the formation of free radicals. Subsequently, these radicals engage with HEMA as the photocurable macromonomer, generating the active substance responsible for propagation. Throughout this propagation phase, crosslinking occurred progressively, resulting in the formation of a three-dimensional (3D) polymer network based on HEMA (Bae et al., 1991).

**FIGURE 3:**
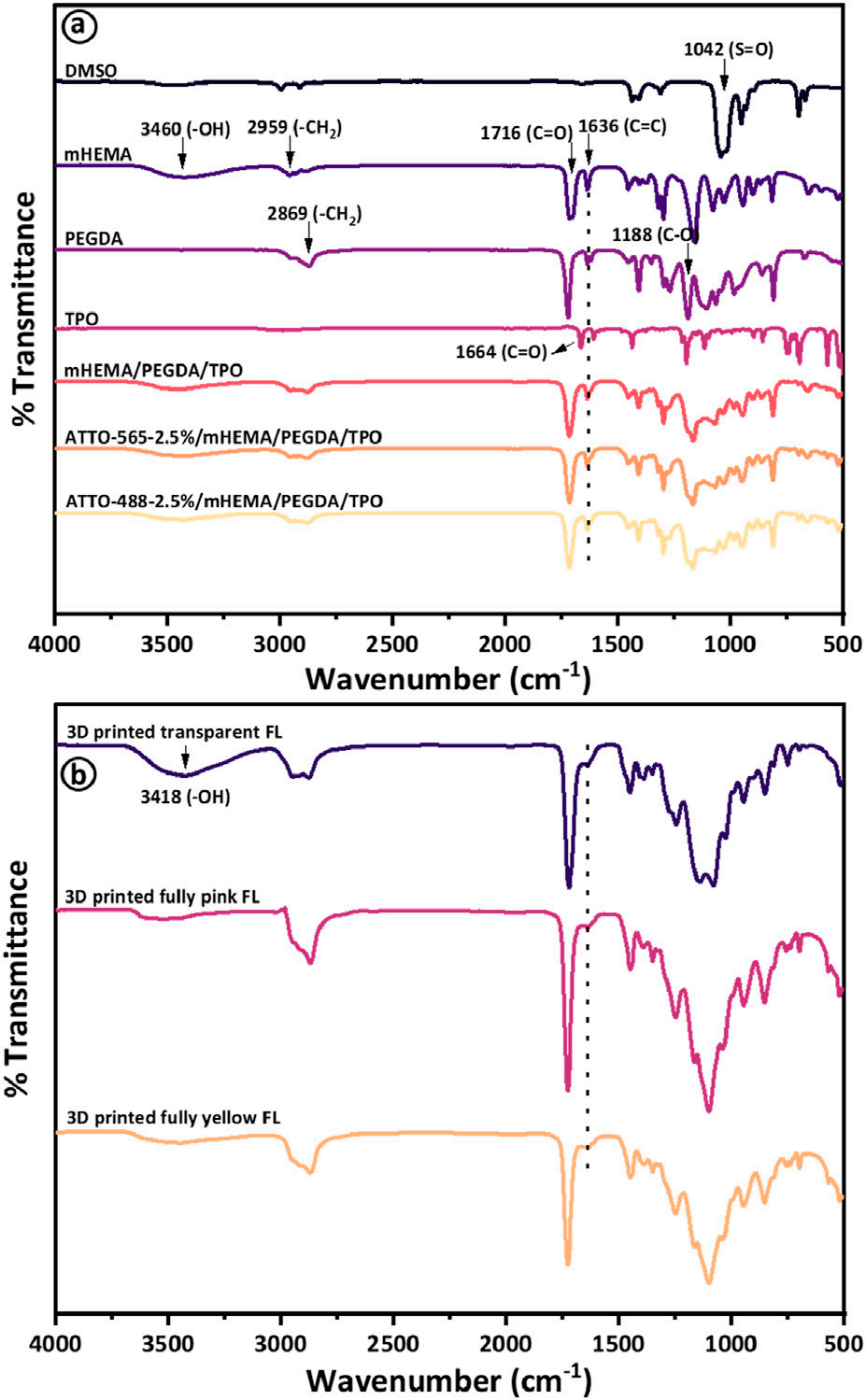
Presents the results of **(A)** Fourier transform infrared (FTIR) spectroscopy analysis of DMSO, HEMA, PEGDA, TPO, and the resultant liquid mixtures containing 2.5 vol% of Atto 565 and Atto 488 dyes before photopolymerization via 3D printing, while **(B)** presents FTIR spectra of respective transparent, fully pink (containing 2.5 vol% of Atto 565), and fully yellow (containing 2.5 vol% of Atto 488) Fresnel lenses after 3D printing process.

FTIR spectra were obtained to identify individual components (DMSO, mHEMA, PEGDA, and TPO) and confirm the polymerized state of HEMA (pHEMA) before and after the printing process (Figure 3). Specifically, Figure 3A includes spectra for DMSO, HEMA, PEGDA, TPO, and the resultant liquid mixtures containing 2.5 vol% of Atto 565 and Atto 488 dyes. These spectra provide insights into the chemical interactions within the mixtures before they undergo 3D printing. Figure 3B presents the FTIR spectra of the fully printed and photopolymerized Fresnel lenses. The lenses are distinguished by their colors: one is fully pink, containing 2.5 vol% of Atto 565 dye, and the other is fully yellow, containing 2.5 vol% of Atto 488 dye. A distinct peak at 1,042 cm^-1^ was observed in the DMSO spectrum, signifying its association with methionine sulfoxide. This peak is attributed to S=O stretching vibrations and aligns well with existing literature (Ravi et al., 2011; Zhang et al., 2017). Previous studies have identified four peaks at 1,636 cm^-1^, 1716 cm^-1^, 2,959 cm^-1^, and 3,460 cm^-1^ for the HEMA monomer (mHEMA), corresponding to C=C, C=O, CH_2_, and -OH bonds, respectively. The 1,636 cm^-1^ peak, indicative of the C=C bond, is present in both HEMA and PEGDA, presented by a dotted line (Figure 3A). Additionally, peaks at 1,188 cm^-1^ and 2,869 cm^-1^ corresponding to C-O and CH_2_ (methylene), respectively, for PEGDA have been reported (Hsueh et al., 2017). A shifted carbonyl band at 1,664 cm^-1^ was also observed for TPO as well. The UV absorption of TPO in the 380–425 nm range with a maximum value at 400 nm demonstrates excellent suitability for precise part printing with full mechanical strength on a 405 nm 3D printer. To achieve high rates of double-bond conversion, a substantial concentration of photoinitiator is imperative (Miletic and Santini, 2012; Steyrer et al., 2017; Yu et al., 2022). Thus, in the 3D printing process, the UV light from the printer activates the photoinitiator (TPO), generating two free radicals (Supplementary Figure 2). These radicals attack the carbon-carbon double bonds in the PEGDA crosslinker, forming a crosslinked network. Simultaneously, the free radicals bond with the vinyl group in mHEMA, further reacting with the crosslinking network to initiate pHEMA/PEGDA polymerization. This process causes the C=C double bonds in mHEMA and PEGDA at 1,636 cm^−1^ to disappear, indicating successful UV photopolymerization through 3D printing. Despite the addition of Atto dyes 565 and 488, no discernible changes representing chemical interactions were observed in their respective FTIR spectra, as depicted in Figure 3A. FTIR spectra following 3D printing of polymerized samples, including transparent and tinted Fresnel lenses, are illustrated in Figure 3B. Notably, the C=C peak at 1,636 cm^-1^ vanished from HEMA and PEGDA, indicating successful synthesis through the UV crosslinking reaction (Hsueh et al., 2017). Similar findings of the photopolymerization process of HEMA-based polymer during 3D printing are reported in (Ali et al., 2024). Moreover, no extra peaks were observed after the printing process to observe chemical interactions; however, the hydroxyl group (-OH) at 3,418 cm^-1^ is still present even after the 3D printing process as shown in Figure 3B (Malik et al., 2023). The FTIR spectra in both subfigures (3a and 3b) provide critical information about the chemical structures and changes that occur during the transition from liquid mixture to solid Fresnel lens, which is essential for understanding the material properties of the final 3D printed products.

**FIGURE 4:**
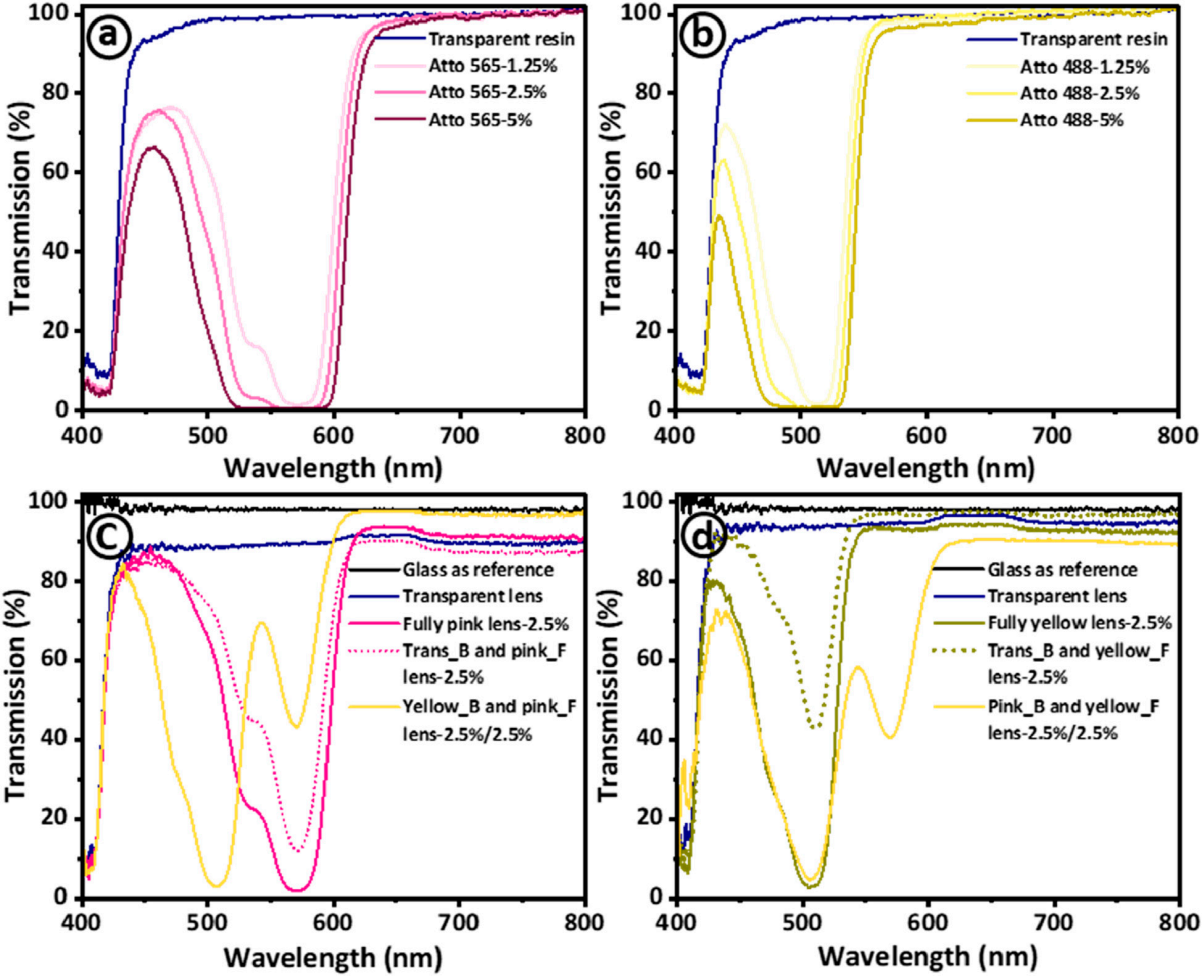
Investigation of optical transmission properties of the materials used in 3D printing of Fresnel lenses designed for addressing color vision deficiency. The transmission spectra are presented for both transparent resin and resin mixtures with varying concentrations (1.25%, 2.5%, and 5%) of **(A)** Atto 565 dye and **(B)** Atto 488 dye. Insets show digital images of Atto dye 565 and Atoo 488 dye. Additionally, the transmission spectra of 3D printed transparent and multifunctional tinted Fresnel lenses are presented with optimized dye concentration of 2.5 vol% for **(C)** Atto 565 and **(D)** Atto 488.

Atto 565 and Atto 488 exhibit a physical appearance of pink and yellow-green (also referred to as chartreuse), respectively. The selection of Atto 565 and Atto 488 is based on their ability to efficiently block undesired wavelengths within the visible spectrum for individuals with CVD. To be more specific, Atto 488 eliminates wavelengths ranging from 480 to 510 nm, while Atto 565 filters out wavelengths between 550 and 580 nm. These dyes have previously demonstrated their effectiveness in addressing problematic wavelengths for CVD patients, as evidenced in prior reports on the production of tinted glasses, contact lenses, and intraocular lenses (Alam et al., 2022b; Alam et al., 2022c; Alam et al., 2023; Hisham et al., 2023). Therefore, when Atto dyes (565 and 488) were mixed with transparent HEMA resin, stable solutions were formed, and similar observations of no chemical interactions can be confirmed from FTIR analysis (Figure 3A). The optical transmission results demonstrate that the transparent HEMA resin exhibits a transmission spectrum surpassing 92% across the entire wavelength range of 400–800 nm. This affirms the appropriateness of choosing the HEMA resin material. The transmission spectra of both dyes were recorded within the visible spectrum range of 400–800 nm with varying concentrations of 1.25%, 2.5%, and 5% by volume (Figures 4A, B). The transmission results indicate sharp transmission dips at ∼570 nm and ∼514 nm for Atto 565 and Atto 488 dyes, respectively (Figures 4A, B). In addition to this, another transmission dip can be observed at ∼413 nm for both dyes (565 and 488). The appearance of transmission dip at ∼413 nm could be attributed to the presence of a photoinitiator (TPO), which absorbs UV light in the 380–425 nm range. Thus, a similar transmission dip was also observed for the transparent HEMA resin solution without the addition of these dye materials (Alam et al., 2022c).

Moreover, as the concentration of both dyes increases from 1.25 to 5 vol% (Figures 4A, B), there is a concurrent widening and strengthening of the transmission dip. Specifically, for Atto 565, the dip’s width at 50% average transmission expanded as wavelength windows of 510–600 nm, 494–605 nm, and 480–611 nm for concentrations of 1.25%, 2.5%, and 5% vol%, respectively. Similarly, for Atto 488, as concentrations increase (1.25, 2.5, and 5 vol%), the observed wavelength windows also expanded as 463–539 nm, 450–536 nm, and 433–534 nm. Furthermore, the intensity of transmission dips for both dyes increase with increasing concentrations, consistently staying below 2%. This demonstrates the effective filtering of these specific wavelengths. It is important to note that transmission intensity is also contingent on the sample’s thickness, gradually diminishing with increased sample thickness (Hamp et al., 2021; Hisham et al., 2023). The inset images in Figures 4A, B represent both dyes along with their corresponding concentrations. Moreover, to maintain adequate optical properties of Fresnel lenses (while preserving transparency), a concentration of 2.5 vol% for both dyes was employed for subsequent stages of 3D printing, material characterization, and testing.

The optical transmission spectra of the printed samples are presented where a glass slide serves as the reference (Figures 4C, D). Figure 4C categorizes the printed samples into the following: transparent lens (Fresnel lens), fully pink lens, Trans_B and pink_F (transparent base and pink features), and Yellow_B and pink_F (yellow base and pink features). The latter two are identified as multimaterial samples, printed in two stages. First, the lens base is printed (with a printer pause for material change and cleaning), followed by the printing of features on the same base to achieve the desired multimaterial Fresnel lenses. As anticipated, there is a slight reduction in the optical transmission of the transparent Fresnel lens post-3D printing. This decline is attributed to changes in the bonding/polymerization of polymeric chains as the material transitions from a liquid to a solid state. However, even after 3D printing, the average transmission of transparent Fresnel lenses remains approximately 90%. The average intensities of transmission dips for fully pink and Trans_B and pink_F lenses are measured at 1.7% and 12.5%, respectively. The 11% decrease in dip intensity of the Fresnel lens (Trans_B and pink_F) is ascribed to the transparent base. Additionally, the transmission windows were found to be 512–594 nm and 530–592 nm, respectively, with a slight decrease observed with the transparent base. The multimaterial sample with a yellow base and pink features (Yellow_B and Pink_F) was printed to combine the wavelength filtering effects of both dyes. Interestingly, two distinct transmission dips at 506 nm and 570 nm were observed for Atto 565 and Atto 488, respectively. Importantly, there were no adverse effects on the multi-material sample during the 3D printing process, underscoring the capability of our 3D printing method to fabricate functional optical devices. However, further improvements in transparency can be obtained by combination of utilized dyes with quantum dots to fabricate higher quality printed optics (Kumar et al., 2017; Malik et al., 2023; ul Gani Mir et al., 2022).

A comparable optical analysis was conducted for tinted Fresnel lenses using Atto 488 (Figure 4D). A similar reduction in transmission dip intensity was noted for the Fresnel lens with a transparent base and yellow features (42.5%) when compared to the fully yellow counterpart (2.9%). Likewise, in the multi-material sample with a pink base and yellow features, two distinct transmission dips were identified at 507 nm and 572 nm, respectively. The transmission dips for all the printed samples matched well with the liquid resin solutions, indicating the effective fabrication of the 3D printing method, even in the case of multimaterial samples. In summary, the printed samples exhibited outstanding optical transmission properties which can be attributed to the optimized layer-by-layer 3D printing process and the smooth surface finish. This minimized losses, specifically in terms of light scattering, owing to the complete diffusion of resin layers during photopolymerization.

**FIGURE 5:**
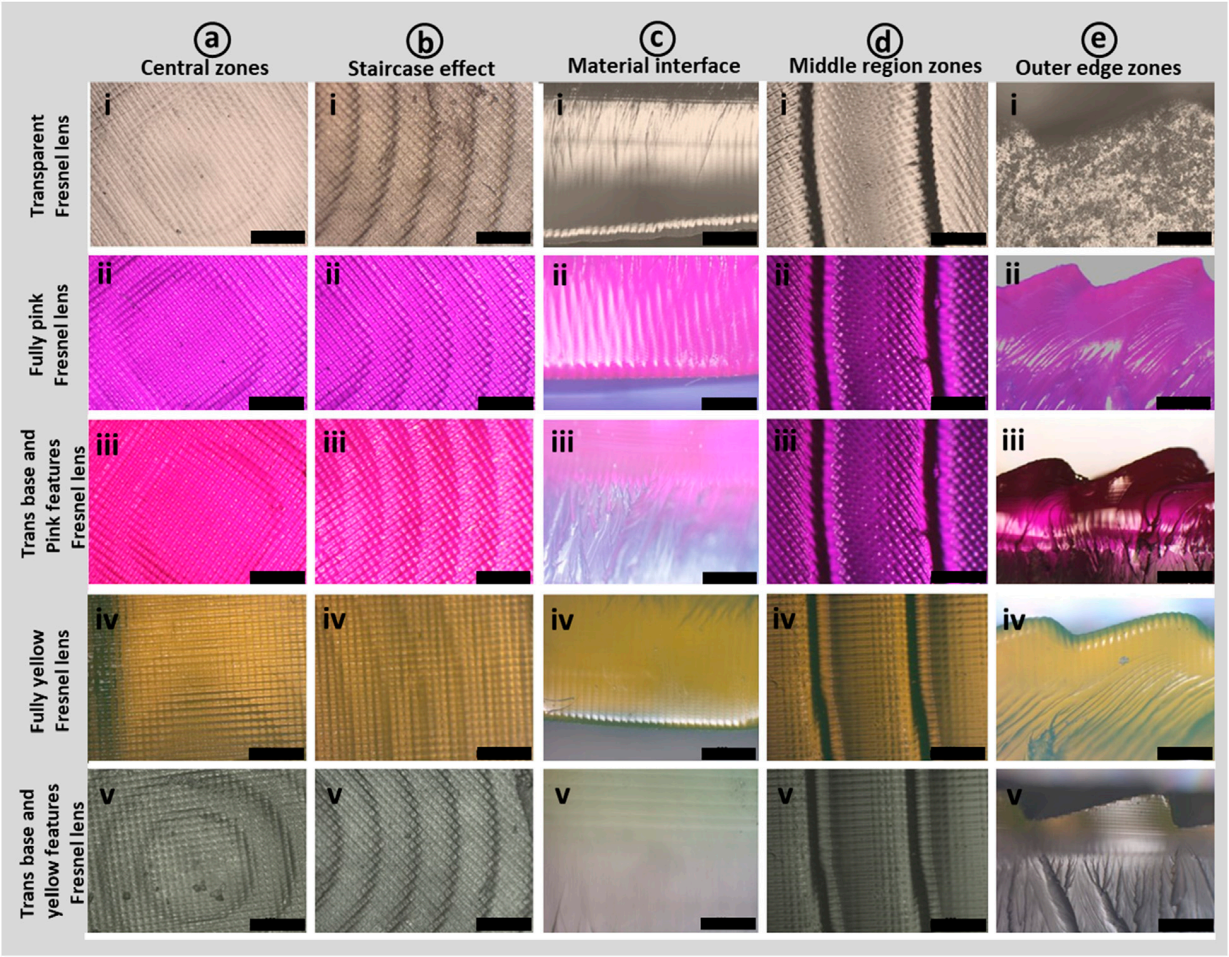
Optical microscopic images present distinct sections of 3D-printed transparent and multifunctional tinted Fresnel lenses. In **(A)**, digital images highlight the central zones or features, encompassing i) transparent lens, ii) fully pink lens, iii) transparent lens with pink features, iv) fully yellow lens, and v) transparent lens with yellow features. Likewise, in **(B–E)** the image set from (i–v) illustrates **(B)** the staircase effect near central zones, **(C)** cross-sectional views, **(D)** middle zones, and **(E)** prismatic features of lenses. All scale bars represent 500 μm.

**FIGURE 6:**
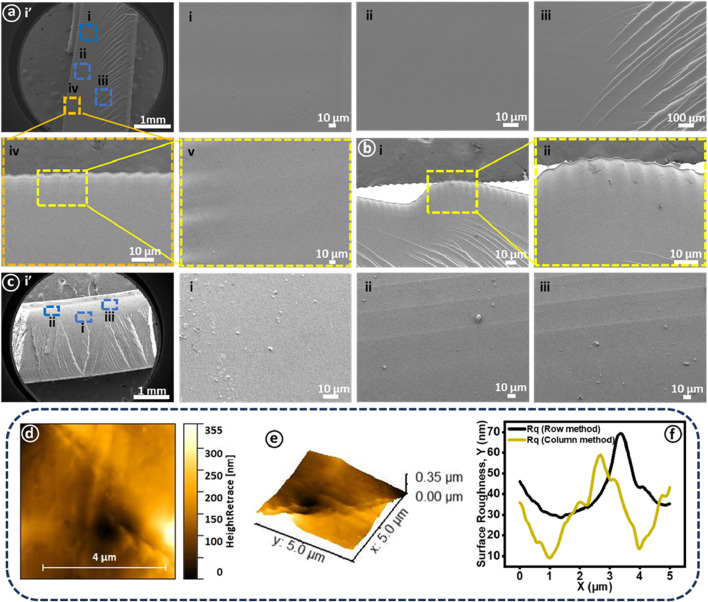
The morphological characterization of 3D-printed lenses was performed using scanning electron microscopy. **(A)** (i) presents transparent lens cross-sectioned sample at low magnification and higher working distance. **(A)** (i-iii) shows cross-sectional views of the printed sample exhibiting smooth surface morphology along with fibrillation while a (iv-v) presents staircase effect near the central zone. **(B)** (i-ii) highlights the staircase effect on prismatic features along with a corresponding high-magnification view. **(C)** (i’) presents cross-sectioned sample modified with Atto 565 at low magnification and higher working distance. **(C)** (i-iii) SEM images of three spots near the base of the lens. Surface roughness measurements of the 3D-printed transparent Fresnel lens encompass **(D)** surface morphology, **(E)** 3D image of the corresponding surface morphology, and **(F)** surface roughness determined through both row and column approaches.

The morphological characterization of the printed Fresnel lenses was conducted by optical microscope in reflection mode as illustrated in Figure 5. The investigated lenses include transparent, fully pink, transparent base with pink features (multimaterial), fully yellow, and transparent base with yellow features (multimaterials), as presented in Figure 5. Optical images of various regions of the printed Fresnel lenses, such as central zones, near-center zones, and outer edge zones are shown in Figures 5A, B, E, respectively. Moreover, the staircase effect near the central zone and the material interface developed between transparent and dye-mixed cured resins are presented in Figures 5B, E, respectively. Overall, the vat photopolymerization based 3D printing process successfully produced both single and multimaterial lenses accompanied by the pixelated effect on the printed surfaces, particularly evident in Figures 5A, B, D. This effect arises because the liquid crystal display (LCD) screen of the printer consists of pixels that activate during UV exposure to print each layer of resin. Figure 5C provides cross-sectional views of the various printed Fresnel lenses, revealing a clear visible interface for multimaterial samples without any discernible voids, indicating the successful integration of the two materials. Furthermore, Figure 5E presents prismatic features of the Fresnel lens for both single and multimaterial Fresnel lenses, along with visible interfaces for multimaterial counterparts. As mentioned previously, the success of 3D printing is closely tied to the optimization of printing parameters and the resulting quality of the produced parts. Therefore, SEM characterization of the tinted sample was conducted to explore surface morphology and provide an in-depth visualization of the layer-by-layer printing process. The cross-sectional views of cryogenically fractured samples are depicted in Figures 6Ai–v. In Figure 6Ai, a low-magnification SEM image of the entire sample is presented, with specific regions marked by square boxes for morphological investigation. Figures 6Ai, ii shows relatively high-magnification images of the smooth surface morphology of printed samples without any artifacts. Consequently, no visible discontinuities or porosity are observed in the printed samples, which can be attributed to the optimized printing parameters. A further magnified SEM image illustrates the precise location where crack growth ceased during the cross-sectioning of the samples. Within this image, the observable ductile yielding and subsequent fibrillation of the fractured sample serve as a clear indication of the material transitioning between brittle and ductile states as shown in Figure 6Aiii (Zotti et al., 2016). The layer-by-layer printing process makes it challenging to produce curved surfaces, leading to the staircase effect, which may impact the overall quality of the printed parts. However, this effect could be minimized by reducing layer thickness or coating the parts with the same resin and then curing them in a UV crosslinking chamber. In this context, a similar staircase effect was observed in the center zone of the lens from both low and high-magnification images (Figures 6Aiv, v). Likewise, the staircase effect was also noted in other zones away from the center as depicted in images (Figures 6Bi, ii).

Moreover, a multimaterial sample (Atto 565) was investigated for surface morphology, with regions of interest marked by rectangular boxes as illustrated in Figure 6Ci. Here, once again, a smooth surface morphology without any visible voids or cracks was observed as shown in Figure 6Ci. However, interestingly, the printing layers were observed in the mixing region as presented in Figures 6Cii,iii. Achieving complete avoidance of the mixing of the two materials during the printing process is indeed a formidable challenge. Despite a comprehensive cleaning procedure during material transitions, a minor degree of mixing persists. In cases where cleaning is not executed appropriately, the mixing phenomenon can intensify, resulting in the latter portion of the print containing traces of the initial material. Additionally, SEM images of the cross-sectioned sample reveal no discernible physical grain boundary or interface between the two printed materials (Hisham et al., 2023).

Surface roughness stands as a critical parameter in evaluating the quality of printed optical components. Higher surface roughness leads to increased light scattering, which proves unfavorable for the optimal functioning of optical devices such as lenses. Typically, optical devices undergo various post-processing treatments alongside optimized printing parameters to enhance surface roughness. These treatments encompass mechanical abrasive polishing, fluid jet polishing, and lacquering (dip coating with the same resin). However, these processes can induce shape deviations and are time-intensive (Heinrich, 2021). In this study, we applied PVC film to the print bed’s surface to print the desired Fresnel lenses. This approach yielded excellent surface smoothness without necessitating any post-processing techniques. The characterization of the printed tinted sample (Atto 565) was conducted using AFM as depicted in Figures 6D–F. A 5 by 5 micron surface area was scanned for which 2D and 3D AFM images are shown in Figures 6D,E. Surface roughness (R_q_) represented by the root mean square (RMS) value was measured using two statistical approaches (row and column), with the respective curves depicted in Figure 6E. The average RMS values were determined to be 52.17 ± 17.19 nm and 39.17 ± 14.07 nm for row and column approaches, respectively. These values underscore the excellent surface smoothness achieved in this work. Notably, the attained results align with the acceptable surface roughness standards (λ/4 to λ/10) for 3D fabricated optical components, where λ represents the curing wavelength (Blachowicz et al., 2021).

**FIGURE 7:**
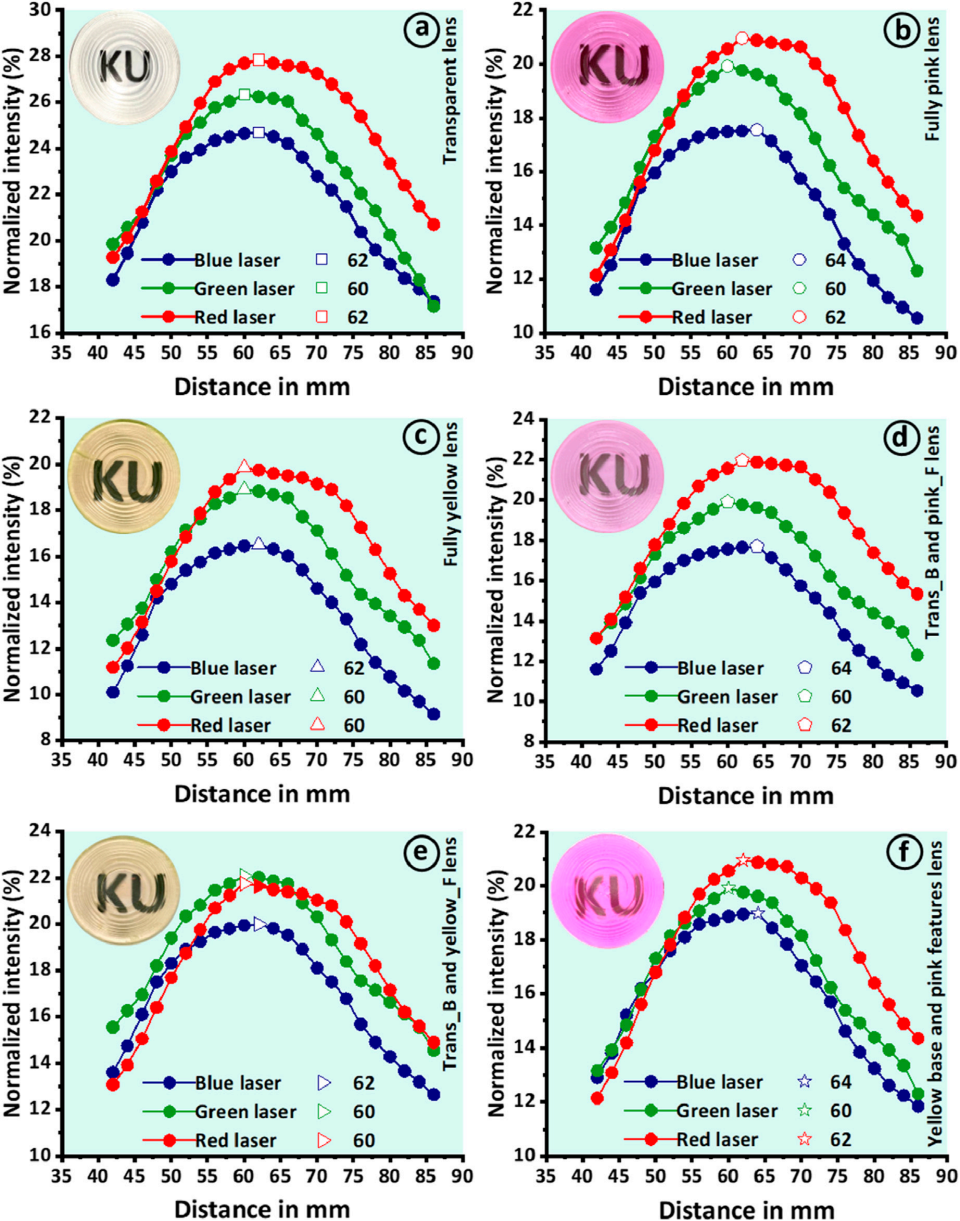
Presents the evaluation of light focusing performance conducted through a customized optical setup for measuring focal lengths. Three lasers (blue, green, and red) were employed to measure the focal lengths of **(A)** transparent lens, **(B)** fully pink lens, **(C)** fully yellow lens, **(D)** transparent lens with pink features, **(E)** transparent lens with pink features, and **(F)** lens with yellow base and pink features. The insets present corresponding 3D-printed Fresnel lenses.

The light focusing performance of high diopter (−16.6 D) transparent and tinted Fresnel lenses was assessed using a customized optical setup comprising monochromatic laser sources, a beam expander, a power meter, and kinematic mounts. Three distinct monochromatic laser sources with wavelengths of 450 nm (blue), 532 nm (green), and 650 nm (red) were employed in this experiment (Supplementary Figure 3). The monochromatic light was expanded using a beam expander, and the printed lenses were then exposed to this expanded beam. The transmitted light through the Fresnel lenses converged to a specific point, known as the focal point. At this focal point, the optical power meter was precisely adjusted on the optical bench, and the light intensity was measured. The measured light intensity was normalized with the power of each laser source and reported as intensity (%) as depicted in Figures 7A–F.

The transparent lens demonstrated effective convergence of incident light for each laser source, resulting in three distinct focal lengths of 60 mm, 62 mm, and 60 mm for the blue, green, and red lasers, respectively. The transparent lens exhibited excellent focusing performance, producing focal points with an average deviation of only 2 mm from the design value of 60 mm (Figure 7A). The tinted lenses also demonstrated strong performance, yielding focal points of 62 mm, 60 mm, and 60 mm for fully pink and 64 mm, 60 mm, and 62 mm for fully yellow when exposed to blue, green, and red lasers, respectively (Figures 7B, C). Notably, the tinted lenses exhibited effective performance, resulting in maximum deviation of 4 mm from the design value. Similarly, multimaterials Fresnel lenses were assessed for their focusing performance using the same methodology. The measured focal lengths were 62 mm, 60 mm, and 62 mm for the lens with a transparent base and pink features (Trans_B and pink_F), 62 mm, 60 mm, and 60 mm for the lens with a transparent base and yellow features (Trans_B and yellow_F), and 64 mm, 60 mm, and 62 mm for the lens with a yellow base and pink features (Yellow_B and pink_F) when tested against blue, green, and red lasers, respectively (Figures 7D–F). Overall, all the lenses demonstrated strong focusing performance, with an average deviation of less than 2 mm. The focusing performance of these lenses is of high importance in this work. This additional functionality of 3D-printed Fresnel lenses make them promising candidates for various eye syndromes such as higher myopia in conjunction with color blindness management. Moreover, its applications may extend beyond myopia, potentially offering benefits in treating various other ocular conditions. Truong Vu et al. reported a high diopter (-5D) diffractive Fresnel lens as an effective tool for treating myopic vision (Vu et al., 2022). These findings suggest that our proposed Fresnel lens could be a versatile tool in ophthalmic care, warranting further investigation into its broader applications in eye disease management.

**FIGURE 8:**
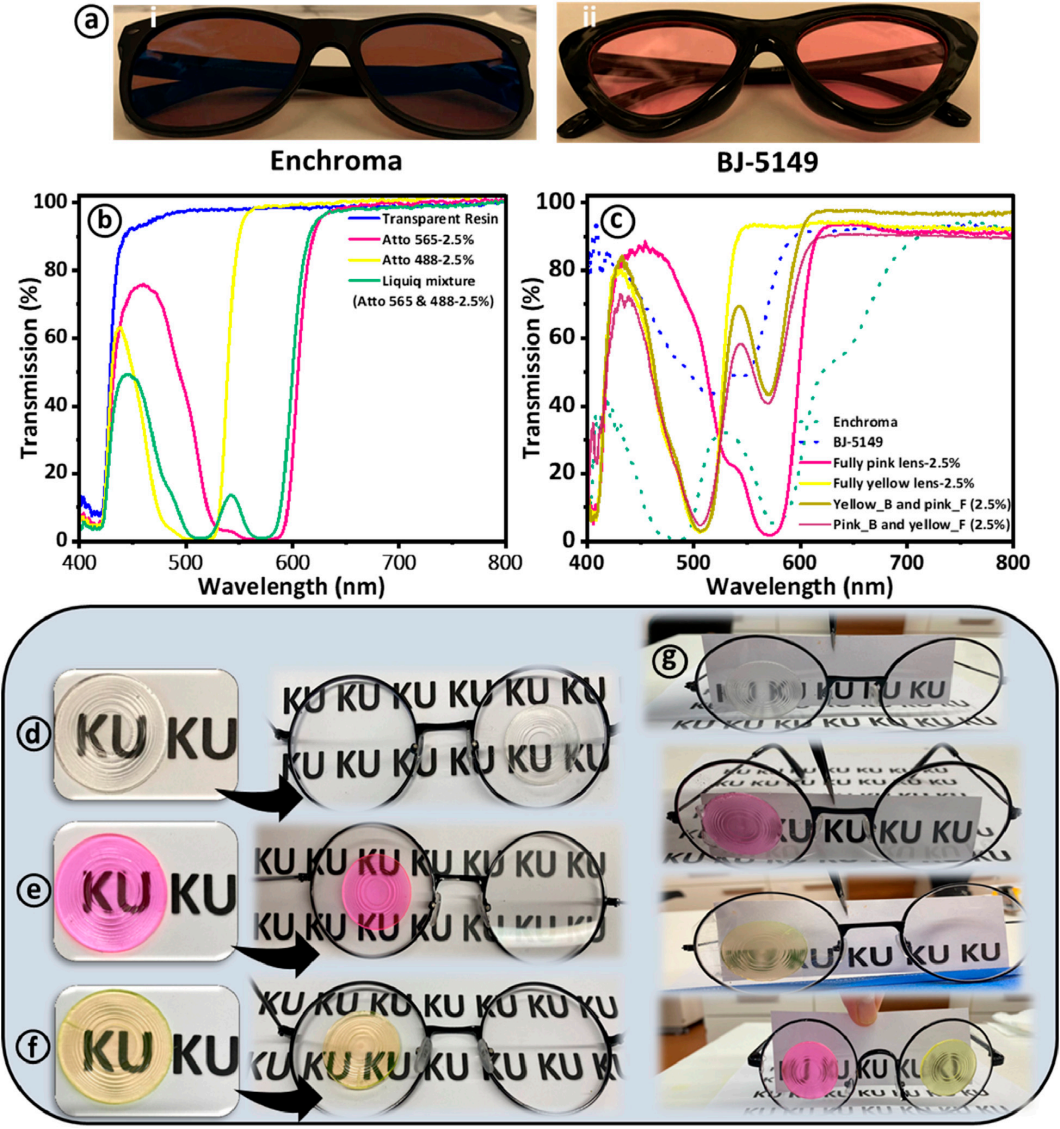
Presents the efficiency comparison between Atto-dyed 3D-printed Fresnel lenses and two commercially available colorblind glasses. **(A)** Presents digital images of commercially available glasses designed for colorblindness management. **(B)** Shows optical transmission spectra of liquid resin before and after the incorporation of 565 and 488 Atto dyes. **(C)** Comparison of transmission spectra of commercial glasses (EnChroma and BJ-5149) with various 3D printed Fresnel lenses **(D–F)** Show digital images of Atto dye-tinted Fresnel lenses and their application onto commercial glasses for image observation. **(G)** Presentation of images observed through tinted Fresnel lenses applied to glasses in a laboratory environment.

The optical transmission properties and wavelength filtering capabilities of 3D-printed tinted Fresnel lenses are compared with commercially available glasses designed for color blindness management in CVD patients, as illustrated in Figure 8. Two specific commercial glasses, namely, EnChroma and BJ-5149, were employed and juxtaposed with our 3D-printed Fresnel lenses (Figures 8Ai,ii). To facilitate a comprehensive analysis, the optical transmission spectra of Atto 565, Atto 488, and their mixture, each constituting 2.5% by volume, are depicted in Figure 8B. In addition to showcasing transmission dips at ∼570 nm and ∼514 nm for Atto 565 and Atto 488, respectively, similar peaks at 571 nm and 512 nm emerged when both dyes were combined in a resin solution at an equal volume percentage of 2.5%. The transmission spectra of the commercial glasses Enchroma and BJ-5149 are meticulously documented and presented in Figure 8C, allowing for a detailed comparison with fully pink, fully yellow, Yellow_B and pink_F, and Pink_B and yellow_F, respectively.

It is discernible that the transmission spectra of our 3D-printed Fresnel lenses exhibit remarkable similarities in certain aspects to those of EnChroma and BJ-5149. These lenses effectively filter out light at comparable wavelengths to their commercial counterparts. Furthermore, the wavelength filtering range of 3D-printed lenses is notably narrower than that of commercial glasses, underscoring their capacity for more selective use in aiding CVD patients. The 3D-printed lenses permit light transmission outside the blocking spectrum, potentially facilitating color differentiation for CVD patients. In contrast, EnChroma and BJ-5149 exhibit more extensive light blocking beyond their designated filtering regions. The obtained results are in close agreement with the previous studies for 3D-printed glasses and contact lenses (Alam et al., 2022c; Alam et al., 2023; Hisham et al., 2023).

The 3D-printed Fresnel lenses, including transparent, fully pink, and fully pink, were also applied to the commercially available glasses to further assess their utilization in potential applications. The pink Fresnel lens can potentially filter out the unwanted light wavelengths for red-green patients, while yellow Fresnel lenses can filter out the unwanted wavelengths for blue-yellow CVD patients. Moreover, the tinted Fresnel lenses were affixed to standard glasses using a thin layer of UV-cured transparent resin for observational purposes. As a result, images in close proximity to the Fresnel glasses are distinctly visible (see Figures 8D–F). A paper sheet was positioned 20 cm in front of the Fresnel glasses, a distance deemed suitable for near vision and conducive to reading. However, the clarity of the images is not as pronounced when compared to commercial glasses, as illustrated in Figure 8G. This discrepancy can be attributed to the dispersion and refraction of light at the grooves and edges of the Fresnel lenses, leading to blurred images (Vu et al., 2022). Nevertheless, images become clear when the capturing camera is positioned close to the transparent and tinted Fresnel lenses.

## 4 Conclusion

In this study, we successfully manufactured multifunctional Fresnel lenses using 3D printing technology. The vat photopolymerization 3D printing process, specifically employing a vat photopolymerization based 3D printer, produced transparent, tinted, and multimaterials Fresnel lenses. The 3D printing UV resin composition consisted of HEMA as the monomer, PEDGA as the crosslinker, and TPO as the photoinitiator. Additionally, the resin was modified with two Atto dyes (565 and 488) known for their ability to filter out problematic wavelengths associated with red-green and blue-yellow colorblindness (550–580 nm and 480–510 nm, respectively). Initially, three different dye concentrations (1.25, 2.5, and 5 vol%) were prepared, but for further characterizations and testing, a 2.5 vol% concentration of each dye was chosen to minimize its impact on the lenses’ transmission properties. Notably, the 3D printing process did not alter the optical properties of the dyes, as confirmed by FTIR characterization showing no chemical interactions between the resin and dyes, with no change in characteristic peaks (transmission dips) before and after printing. SEM characterization of cross-sectioned samples revealed no voids or discontinuities, indicating excellent printing quality and optimized printing parameters. Atomic force microscopy (AFM) results indicated a satisfactory average surface roughness of approximately 52.17 nm and 39.17 nm using row and column methods, respectively. These values fell within the acceptable range of λ/4 to λ/10 for optical devices, attributed to using PVC film on the print bed surface. The multimaterial fabrication of lenses was straightforward, displaying visible material interfaces without impacting optical properties. Focusing performance, evaluated using a customized optical setup, resulted in focal length measurements with less than a 2 mm average deviation from the design values. This added multifunctionality to Fresnel lenses, suggesting their potential application for myopic disorder corrections. Furthermore, a comparison of the transmission properties between 3D-printed Fresnel lenses and commercial glasses revealed that the printed lenses exhibited greater selectivity in blocking unwanted light wavelengths, which is beneficial in CVD correction. These findings underscore the potential applications of multifunctional Fresnel lenses fabricated through a single-step, facile 3D printing process in ophthalmology, demonstrating their effectiveness in vision correction and imaging systems.

## Data Availability

The raw data supporting the conclusions of this article will be made available by the authors, without undue reservation.
