# Supplementary material for: Vat photopolymerization of multifunctional fresnel lenses for ocular management
Source: Front Bioeng Biotechnol. 2024 Oct 8;12:1464129. doi: 10.3389/fbioe.2024.1464129 (PMC11493721; doi:10.3389/fbioe.2024.1464129)
Supplement: Supplementary file 1 [file Table1.DOCX]

**Vat Photopolymerization of Multifunctional Fresnel Lenses for Ocular Management**

Murad Ali^1,2*^, Muhammed Hisham^1^, Rashid K. Abu Al-Rub^1,2^, Haider Butt^1,2*^

*^1^* Department of Mechanical and Nuclear Engineering, Khalifa University of Science and Technology, Abu Dhabi, 127788 UAE


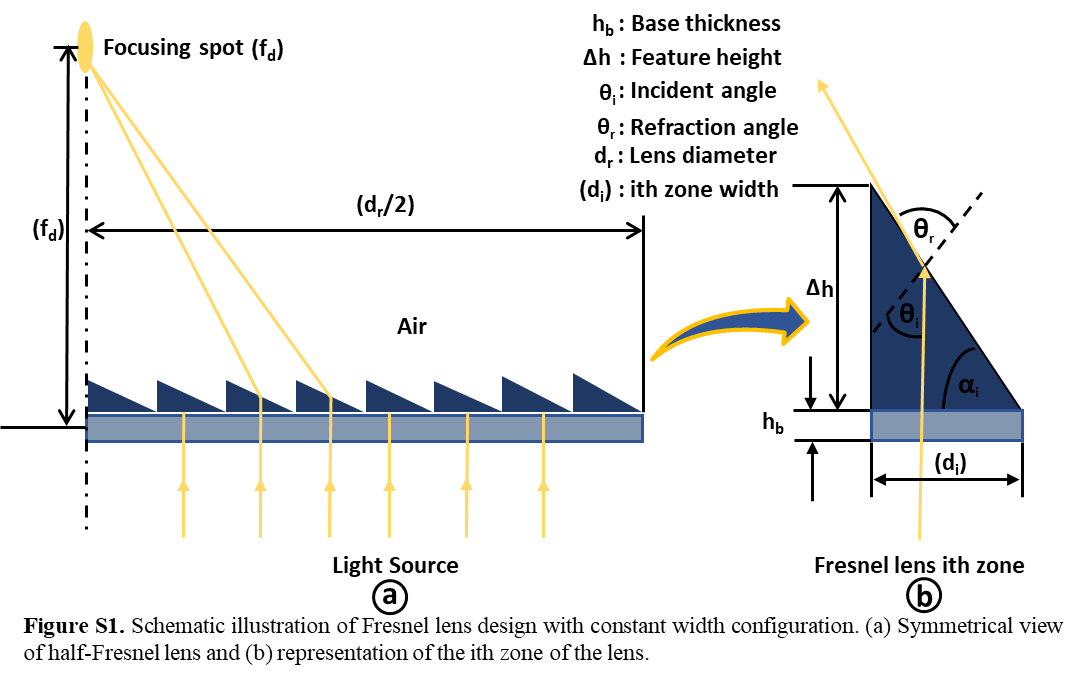
^2^Advanced Digital & Additive Manufacturing (ADAM) Center, Khalifa University of Science and Technology, Abu Dhabi 127788, United Arab Emirates


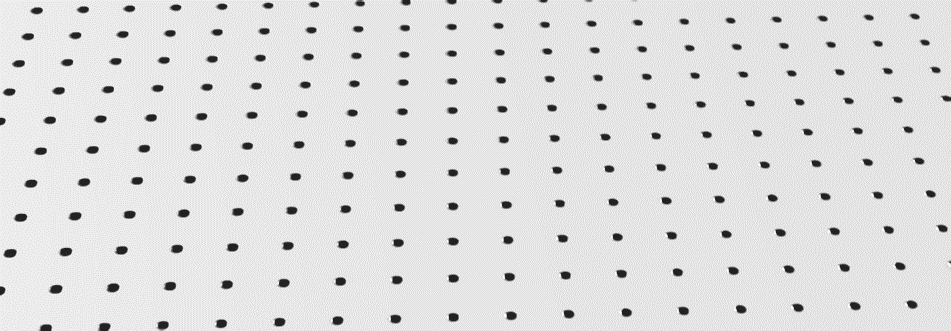

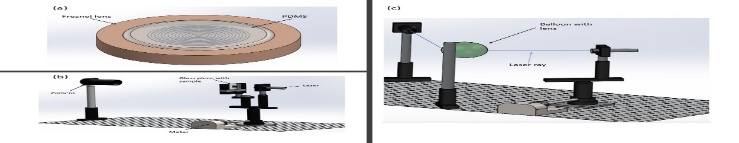


Laser source (532 nm)

Beam expander

3D-printed Fresnel lens

Photodetector

Input laser

beam

Expanded beam

Converging beam


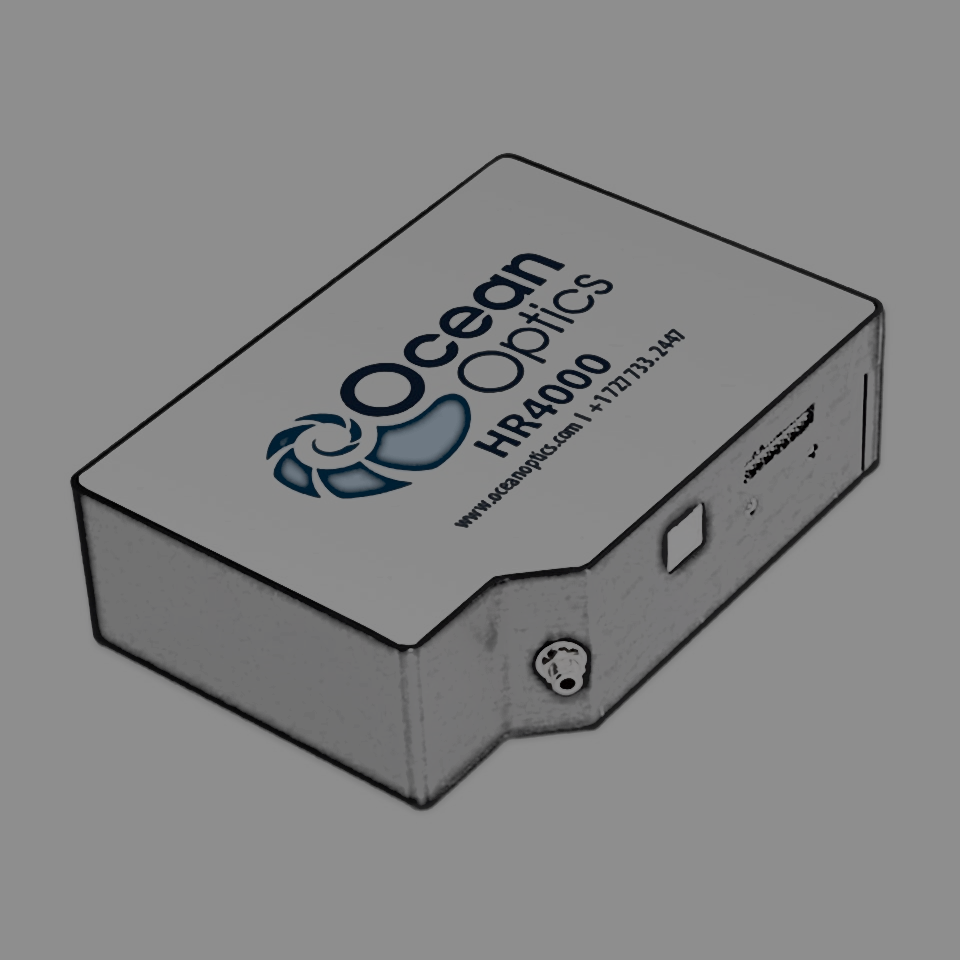

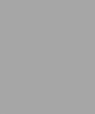

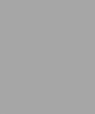

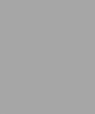

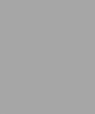

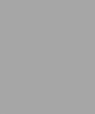

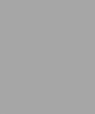

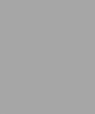

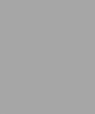

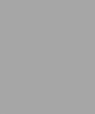

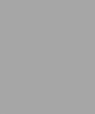

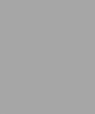


**Figure SI 2**: Schematic illustration of optical setup utilized for focal length measurements.
